# Subclinical and attenuated negative symptoms in the prediction of psychosis in children and adolescents: sample composition matters

**DOI:** 10.1007/s00787-026-03042-2

**Published:** 2026-06-06

**Authors:** Santiago Cabrera, Naweed Osman, Chantal Michel, Maurizia Franscini, Petra Walger, Nina Traber-Walker, Benno G. Schimmelmann, Rahel Flückiger, Volker Reissner, Frauke Schultze-Lutter

**Affiliations:** 1 Department of Child and Adolescent Psychiatry, Psychotherapy and Psychosomatics, LVR Clinic Düsseldorf, Clinics of the Heinrich-Heine University, Düsseldorf, Germany; 2 Department of Child and Adolescent Psychiatry, Psychosomatics and Psychotherapy, Hospital of the Ludwig-Maximilians-University (LMU) Munich, Nußbaumstraße 5a, 80336 Munich, Germany; 3 Department of Psychiatry and Psychotherapy, Medical Faculty, University Hospital Düsseldorf, Heinrich-Heine-University Düsseldorf, Düsseldorf, Germany; 4 University Hospital of Child and Adolescent Psychiatry and Psychotherapy, University of Bern, Bern, Switzerland; 5 Department of Child and Adolescent Psychiatry and Psychotherapy, University of Zürich, Zürich, Switzerland; 6 University Hospital of Child and Adolescent Psychiatry, University Hospital Hamburg- Eppendorf, Hamburg, Germany; 7 Department of Child and Adolescent Psychiatry, Psychotherapy and Psychosomatics, LVR Clinic Essen, University of Duisburg-Essen, Essen, Germany; 8 Department of Psychology, Faculty of Psychology, Airlangga University, Surabaya, Indonesia

**Keywords:** Clinical high risk, Psychoses, Negative symptoms, Basic symptoms, Childhood, Adolescence

## Abstract

In children and adolescents, subjective and attenuated negative symptoms were hypothesized to best differentiate clinical-high risk for psychosis (CHR-P) from other conditions, and to be most predictive of psychosis. To examine this, we analysed data from 8-17-year-old CHR-P in-/outpatients (*n* = 183), and inpatients not clinically suspected of developing psychosis (*n* = 277) based on cross-sectional and two-year naturalistic follow-up data from a Swiss-German study. CHR-P criteria and other symptoms were assessed using the Schizophrenia Proneness Instrument, Child & Youth version, and the Structured Interview for Psychosis-Risk Syndromes. Pairwise comparisons, two-step cluster analyses and Cox regression models were applied to identify cluster profiles and the best predictors of psychosis. In pairwise comparisons, cognitive and perceptive basic symptoms, and attenuated positive symptoms better distinguished CHR-P patients from non-CHR-P patients and converters from non-converters than the negative symptom subscales “Adynamia” and “Negative symptoms. In the stable two-cluster solution of patients scoring high (n = 338) and low (n = 122) on all scales, “Adynamia” was most important (importance = 1.0) whereby the high-scorer cluster included most of CHR-P (86.9%) and converters (84.6%), and was predictive of psychosis. Cox models of subscales differed in the selected predictors depending on the analysed samples (total sample, CHR-P and ultra-high risk only (UHR)), whereby the earlier report of “Negative symptoms” predicting conversion in UHR was replicated when only SIPS subscales were considered. While our findings partially support the significance of subjective and attenuated negative symptoms in CHR-P and psychosis prediction, they also suggest that the role of negative symptoms is highly dependent on sample composition.

**Supplementary Information:**

The online version contains supplementary material available at https://doi.org/10.1007/s00787-026-03042-2.

## Introduction

Psychoses are a leading cause of disability starting from age 10 years, and continuing into adulthood [1]. However, notable developmental differences exist in the clinical presentation of psychosis between minors and adults [2–4]. These developmental aspects also play a crucial role in the early detection of psychosis [3, 5, 6], and may contribute to the observed lower conversion rates to manifest psychosis in minors at clinical high risk for psychosis (CHR-P; note: we use this as an umbrella term for both ultra-high risk (UHR) and basic-symptom criteria) [7, 8]. A potential explanation for this phenomenon is the higher prevalence and lower clinical relevance of symptoms used in CHR-P criteria in underage community subjects [9–11].

Another potential factor contributing to the age-related differences in transition rates may be the varying clinical significance for conversion of other symptom domains relevant to the diagnosis of psychoses, such as negative symptoms (NS) [12–17]. NS include five phenomena, mainly assessed by observations [18]: blunted affect, alogia, anhedonia, asociality and avolition. Before NS become observable to others, they may be self-experienced as subtle disturbances in mental processes such as drive, volition, stress tolerance, emotional responsiveness and processes, in terms of so-called *adynamic* basic symptoms (BS) [14]. Subjective and observable NS frequently precede the development of positive symptoms, and are associated with important social and role dysfunction [15–17].

Due to their high prevalence in truly prodromal and CHR-P states, attenuated NS have been proposed as an independent risk syndrome for psychosis onset, particularly in minors [16, 19]. This is supported by studies that indicate a more important role for subjective and attenuated NS in underage compared to adult samples. For instance, in dimensional studies of BS [20, 21], the BS subsumed in the dimension “Adynamia” of the child and youth version of the “Schizophrenia Proneness Instrument” (SPI-CY) [22] formed a central domain in minors, while in adults, the two cognitive dimensions of the adult version (SPI-A) [23] formed the centre. Moreover, more BS of the negative dimension were included in the SPI-CY, while in adults, these often did not correlate sufficiently with their own cluster and were therefore not included in the SPI-A [20]. Finally, in the SPI-CY pilot study on an underage sample [24], “Adynamia” discriminated best between CHR-P patients from patients not suspected to develop psychosis and healthy controls. This suggested that this subscale may play a unique role in the early detection in this age group [24]. Furthermore, a three-year follow-up study identified age-specific prediction models for ultra-high risk (UHR) patients, distinguishing between underage and adult groups [25]. In minors, the “Negative symptoms” subscale of the “Structured Interview for Psychosis-Risk Syndromes” (SIPS) [26] emerged as the strongest predictor of psychosis, whereas “Positive symptoms” best predicted conversion in adults. These findings support the idea that underage and adult CHR patients may benefit from tailored approaches to early detection, with NS potentially playing a pivotal role in minors [24, 25]. Thus, taken together, these initial studies indicate that the reduction of subjective and attenuated NS might be given more attention in future early detection and intervention trials and that future studies should differentially investigate their role in CHR-P states.

### Aim of the study

In this first-time study of both subjective and attenuated NS assessed by the SPI-CY subscale “Adynamia” and the SIPS subscale “Negative symptoms”, respectively (see sText 1 for details), we differentially examined their role in 8- to 17-year-olds in separating the CHR-P state from other clinical states and in predicting psychosis. In line with the SPI-CY pilot study by Fux et al. [24] and prediction study by Zhang et al. [25], it was assumed that “Adynamia” and “Negative symptoms” would best distinguish CHR-P from other clinical states and would be most predictive of a conversion to psychosis.

## Methods

### Sample

We used data from the German-Swiss naturalistic longitudinal “Bi-National Evaluation of At-Risk Symptoms in Children and Adolescents” (BEARS-Kid) study [27], which comprises a total of *N* = 460 with both a clinical control group (CC; *n* = 277) and a CHR-P group (*n* = 183). The BEARS-Kid study was conducted between 09/2013 and 02/2018 in the child and adolescent psychiatric departments of the universities of Bern, Zurich and Cologne. General inclusion criteria were age between 8 and 17 years, sufficient language skills in German, French or English, and IQ > 70. An additional inclusion criterion for the CHR-P group was the presence of any UHR or BS criteria. An additional inclusion criterion for the clinical controls was inpatient or day clinic treatment for any primary diagnoses that had been related to a higher frequency of psychosis in adulthood [28] (sFigure 1), i.e., anxiety (social phobia, specific phobia) and obsessive-compulsive disorder, eating disorders (anorexia and bulimia nervosa), and attention deficit and hyperactivity disorder (ADHD). Furthermore, Asperger’s disorder was included as a primary diagnosis in childhood and adolescence with a higher likelihood of psychosis in adulthood [29].

General exclusion criteria were mental symptoms being due to the direct physiological effect of a medical condition, and lifetime diagnosis of a psychotic disorder. An additional exclusion criterion for the CHR-P was CHR-P symptoms unequivocally being due to the direct physiological effect of a substance. An additional exclusion criterion for the CC was an ongoing antipsychotic treatment, clinical suspicion of an emerging psychosis or presence of any CHR-P criteria; in case of the latter, they were included into the CHR-P group. Furthermore, a third sample was recruited from the community that also contributed some subjects to the CHR-P group (sFigure 1). However, if these community subjects did not fulfil CHR-P criteria, they were not included in the present analyses (see sText 2 for additional details on recruitment).

Patients from CHR-P and CC groups were followed-up for 12 and 24 months for a transition to psychosis. Of the 460 patients, *n* = 137 and *n* = 85 CHR-patients, as well as *n* = 220 and *n* = 173 CC participated in the one-year and two-year follow-up (sFigure 1). Participants with no follow-up data (*n* = 83; 18.0%) were conservatively categorized as “non-converters” for analytical purposes (see sText 3 for details).

The study was reviewed and approved by the ethical committees of the University of Bern (No. 174/10), the University of Zurich (No. 2010 − 0415/3), and the University of Cologne (No. 11–071). All participants, and their legal guardian, provided informed written consent prior to study enrolment.

### Assessments

The BS criteria “Cognitive Disturbances” (COGDIS) and “Cognitive-Perceptive BS” (COPER) (sTable 1a) as well as other BS were assessed with the SPI-CY [21] that includes four subscales: ”Adynamia”, ”Perceptual disturbances”, ”Neuroticism”, and “Thought and motor disturbances”. BS are rated according to their frequency in the past three months using scores ranging from ‘0’ (absent) to ‘6’ (daily). In addition, scores of ‘7’ (trait feature and, consequently, not a basic symptom by definition), ‘8’ (present, but frequency unknown), and ‘9’ (questionable present) can be assigned. Following rating instructions of the SPI-CY [21], we recoded scores of ‘7’ and ‘9’ to ‘0’ and ‘8’ to ‘1’, before calculating the sum scores of the subscales. To assess the three ultra-high risk (UHR) criteria, i.e., the Attenuated Psychosis Symptoms Syndrome (APSS), the Brief Intermittent Psychotic Symptoms Syndrome (BIPSS) and the Genetic Risk and Functional Decline syndrome (GRFD) (sTable 1b), and negative and other symptoms, we used the SIPS [11] that has four subscales: positive, negative, disorganization, and general symptoms. Items are syndromally rated according the quality of affirmed symptoms from ‘0’ (absent) to ‘6’ (severe and psychotic/extreme).

Interviews were conducted by clinical psychologists who had received an intensive three-month training, especially in the assessment of CHR-P symptoms/criteria and mental disorders, in order to achieve a ≥ 95% concordance rate with the trainers (F.S.-L. and C.M.). Weekly supervision of symptom ratings performed by the two trainers further ensured consistently excellent, valid and reliable data quality across centres.

### Data analysis

Data analyses were performed using IBM SPSS Statistics version 29.0. Group differences in sociodemographic and clinical characteristics were examined using Mann-Whitney-U tests with Rosenthal’s r as effect size for continuous variables and Chi²-tests with Cramer’s V as effect size for categorical variables. In addition, receiver operating characteristic (ROC) analyses of the subscales were carried out for groups to explore their overall group-discriminative power.

A two-step cluster analysis procedure was used to identify profiles across the sample and subscales’ importance to the cluster solution using a random order of cases. Because the solution of the two-step cluster analysis can be influenced by the order in which cases enter, the analysis was repeated in four different sorting of cases (groups, age, sex and educational level) to test for the solution’s stability.

Cox regression analyses (stepwise Wald method for subscales and enter with bootstrapping for clusters) were used to explore the psychosis-predictive ability of the subscales and clusters in the UHR, the CHR-P and total sample.

## Results

### Sample characteristics

The CHR-P and CC group differed in age, nationality, level of education and the frequency of most mental disorders (Table 1). Patients with and without any follow-ups differed in their educational level in both groups. In the CHR-P group, patients with and without follow-up assessment showed differences in age, sex and nationality, but not in clinical characteristics (sTable 2, and 3). CHR-P patients scored higher across all subscales compared to CC, with moderate to high effect sizes (Table 2). These differences were reflected in areas under the curve ranging from moderate to excellent, with “Perception disturbances” showing good and “Positive symptoms” showing excellent discriminative performance (sTable 4).

**Table 1:** Baseline characteristics of sample (*N* = 460)

	CHR-*P* (*n* = 183, 39.8%)	Clinical controls (*n* = 277, 60.2%)	Total sample (*N* = 460)	Statistics, effect size
Age, mean (SD), median	15.30 (2.07), 15.73	14.26 (2.54), 14.86	14.68 (2.41), 15.15	z=-4.484, *p* < 0.001;*r*=-0.209
Sex, male, n (%)	69 (37.7%)	126 (45.5%)	195 (42.4%)	χ²(1) = 2.733, *p* = 0.102; V = 0.077
Nationality, n (%)				χ²(2) = 8.719, *p* = 0.013; V = 0.138
Switzerland	102 (55.7%)	153 (55.2%)	255 (55.4%)	
Germany	57 (31.1%)	108 (39.0%)	165 (35.9%)	
Other	24 (13.1%)	16 (5.8%)	40 (8.7%)	
Highest achieved/targeted school graduation, n (%)				χ²(3) = 13.305, *p* = 0.004; V = 0.170
ISCED 0	2 (1.1%)	1 (0.4%)	3 (0.7%)	
ISCED 1	21 (11.5%)	69 (24.9%)	90 (19.6%)	
ISCED 2	89 (48.6%)	116 (41.9%)	205 (44.6%)	
ISCED 3	71 (38.8%)	91 (32.9%)	162 (35.2%)	
Clinical setting, n (%)				χ²(1) = 232.095, *p* < 0.001; V = 0.710
Inpatient/day clinic	68 (37.2%)	277 (100%)	345 (75.0%)	
Outpatient	115 (62.8%)	0 (0.0%)	115 (25.0%)	
COGDIS, n (%)^a^	81 (44.3%)	0 (0.0%)	81 (17.6%)	χ²(1) = 148.810, *p* < 0.001; V = 0.569
COPER, n (%)^a^	114 (62.3%)	0 (0.0%)	114 (24.8%)	χ²(1) = 229.412, *p* < 0.001; V = 0.706
APSS, n (%)^a^	112 (61.2%)	0 (0.0%)	112 (24.3%)	χ²(1) = 224.091, *p* < 0.001; V = 0.698
BIPSS, n (%)^a^	5 (2.7%)	0 (0.0%)	5 (1.1%)	χ²(1) = 7.651, *p* = 0.010; V = 0.129
GRFD, n (%)	10 (5.5%)	0 (0.0%)	10 (2.2%)	χ²(1) = 15.437, *p* < 0.001; V = 0.183
Any affective disorders, n (%)^a, b^	69 (37.7%)	43 (15.5%)	112 (24.3%)	χ²(1) = 29.435, *p* < 0.001; V = 0.253
Any anxiety disorders, n (%)^a, b^	75 (41.0%)	72 (26.0%)	147 (32.0%)	χ²(1) = 11.389, *p* < 0.001; V = 0.157
Any obsessive-compulsive disorders, n (%)^a, b^	15 (8.2%)	31 (11.2%)	46 (10.0%)	χ²(1) = 1.098, *p* = 0.342; V = 0.049
Any eating disorders, n (%)^a, b^	16 (8.7%)	86 (31.0%)	102 (22.2%)	χ²(1) = 31.766, *p* < 0.001; V = 0.263
Any somatoform disorders, n (%)^a, b^	3 (1.6%)	3 (1.1%)	6 (1.3%)	χ²(1) = 0.265, *p* = 686; V = 0.024
Any substance misuse, n (%)^a, b,c^	15 (8.2%)	4 (1.4%)	19 (4.1%)	χ²(1) = 12.690, *p* < 0.001; V = 0.166
Any trauma/stress-related disorders, n (%)^a, b^	13 (7.1%)	2 (0.7%)	15 (3.3%)	χ²(1) = 14.227, *p* < 0.001; V = 0.176
Any developmental disorders, n (%)^a, b^	25 (13.7%)	122 (44.0%)	147 (32.0%)	χ²(1) = 46.780, *p* < 0.001; V = 0.319
SOFAS, mean (SD), median	59.67 (12.08), 60.00	60.25 (11.10), 60.00	60.02 (11.49), 60.00	z=-0.864, *p* = 0.387; *r*=-0.040
Conversion to psychosis, n (%)	12 (6.6%)	1 (0.4%)	13 (2.8%)	χ²(1) = 15.407, *p* < 0.001; V = 0.183

^a^ multiple CHR-P criteria and diagnosis possible

^b^ assessed with the Mini-International Neuropsychiatric Interview for Children and Adolescents (MINI-Kid)

*ISCED*: International Standard Classification of Education 2011; *CHR-P*: clinical high-risk for psychosis; *COGDIS*: Cognitive Disturbances; *COPER*: Cognitive-Perceptive basic symptoms; *APSS*: attenuated psychotic symptoms syndrome; *BIPSS*: brief intermittent psychotic symptoms syndrome; *GRFD*: genetic risk and functional decline; *SOFAS*: Social and Occupational Functioning Assessment Scale (0 = lowest to 100 = highest functioning)

^c^ Please note: If CHR-P symptoms occurred clearly independently of substance effects, for example, by having occurred before the beginning of substance use or by having continued to occur in substance-free periods, they were considered as to not being exclusively the *direct* physiological effect of a substance, thus not meeting the respective exclusion criterion

**Table 2:** Subscale means and cluster distribution of the two groups (*N* = 460)

	CHR-*P* (*n* = 183, 39.8%)	Clinical controls (*n* = 277, 60.2%)	Statistics, effect size	
SPI-CY subscales, mean (SD), median				
Adynamia	29.40 (20.11), 27.00	11.76 (13.19), 8.00	z=-9.643, *p* < 0.001, *r*=-0.450	
Perception disturbances	10.92 (12.34), 8.00	1.16 (2.60), 0.00	z=-12.997, *p* < 0.001, *r*=-0.606	
Neuroticism	9.58 (7.36), 9.00	4.53 (4.77), 3.00	z=-7.645, *p* < 0.001, *r*=-0.356	
Thought and motor disturbances	14.14 (12.63), 12.00	3.42 (4.99), 1.00	z=-10.636, *p* < 0.001, *r*=-0.496	
SIPS subscales, mean (SD), median				
Positive symptoms	8.38 (4.28), 8.00	2.19 (2.26), 1.00	z=-14.774, *p* < 0.001, *r*=-0.689	
Negative symptoms	9.87 (5.79), 10.00	5.30 (4.20), 5.00	z=-8.420, *p* < 0.001, *r*=-0.393	
Disorganization symptoms	3.87 (2.53), 3.00	2.13 (1.69), 2.00	z=-7.491, *p* < 0.001, *r*=-0.349	
General symptoms	8.27 (4.30), 9.00	4.56 (3.44), 4.00	z=-9.134, *p* < 0.001, *r*=-0.426	
Clusters				
Cluster 1, included, n (%)	106 (57.9%)	16 (5.8%)	χ²(1) = 153.772, *p* < 0.001; V = 0.578	

### Cluster analysis

**Fig. 1:**
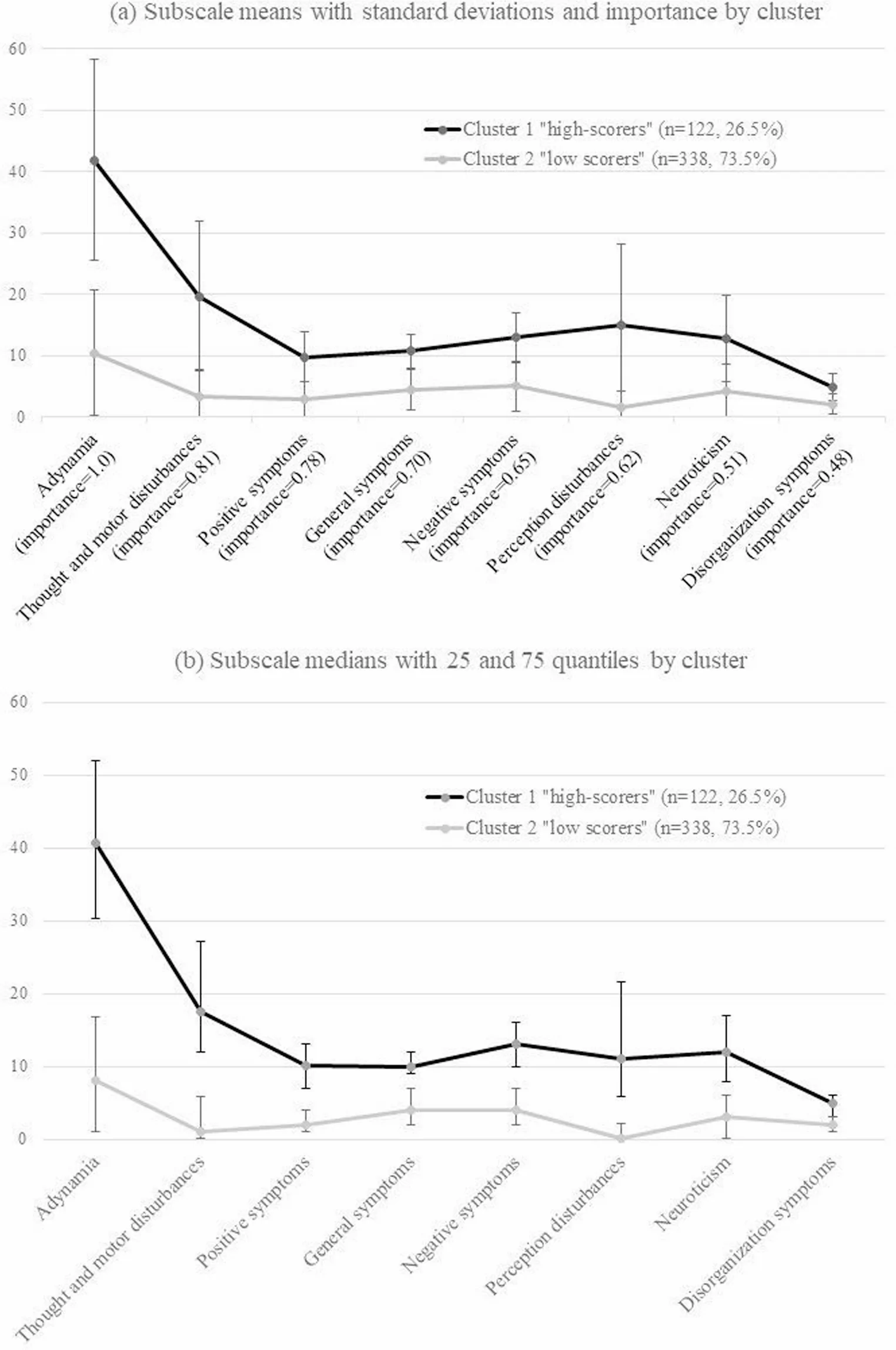
The two-cluster solution; (**a**) means with standard deviations and importance, (**b**) medians with 25 and 75 quantiles

All sortings resulted in the same stable two-cluster solution, separating high- from low-scorers across all subscales (Fig. 1). Silhouette measure of cohesion and separation was 0.5, indicating fair cluster quality. The ratio of largest-to-smallest cluster was 2.77. “Adynamia” was the most important subscale for clustering (Fig. 1).

Cluster 1 included a higher rate of CHR-P than of CC patients (Table 2). Furthermore compared to Cluster 2 patients, Cluster 1 patients were older, mostly female, had higher rates of substance use, affective, anxiety and stress-related disorders but lower rates of eating and developmental disorders, and poorer psychosocial functioning (sTable 5).

### Prediction of psychosis

All but one converter (*n* = 13, 2.8%) belonged to the CHR-P group (Table 1, sTable 6). Converters scored statistically significantly higher in all subscales except “Neuroticism” compared to non-converters, and were mostly part of Cluster 1 (Table 3). The Cox regression models on the subscales of SPI-CY and SIPS selected “Positive symptoms” and “Negative symptoms” in the total sample, “Disorganized symptoms” in the CHR-P sample, and “Negative symptoms” and, negatively, “Neuroticism” in the UHR sample. When only SIPS subscales were considered, only “Negative symptoms” were predictive in the UHR sample (Table 4). In the Cox regression models on the two clusters, Cluster 1 was a significant predictor in all samples, albeit with a large confidence interval (Table 4).

**Table 3:** Subscale means and cluster distribution of converters and non-converters (*N* = 460)

	Non-converters (*n* = 447, 97.2%)	Converters (*n* = 13, 2.8%)		Statistics, effect size	
SPI-CY subscales, mean (SD), median					
Adynamia	18.23 (18.05), 13.00	37.46 (22.44), 46.00		z=-3.063, *p* = 0.002, *r*=-0.143	
Perception disturbances	4.72 (8.91), 0.00	16.23 (15.73), 7.00		z=-3.970, *p* < 0.001, *r*=-0.185	
Neuroticism	6.46 (6.40), 6.00	9.23 (7.12), 8.00		z=-1.532, *p* = 0.126, *r*=-0.071	
Thought and motor disturbances	7.28 (9.94), 4.00	21.46 (12.79), 20.00		z=-4.277, *p* < 0.001, *r*=-0.199	
SIPS subscales, mean (SD), median s					
Positive symptoms	4.48 (4.31), 3.00	10.46 (4.35), 10.00		z=-4.275, *p* < 0.001, *r*=-0.199	
Negative symptoms	6.94 (5.23), 6.00	13.38 (6.73), 17.00		z=-3.414, *p* < 0.001, *r*=-0.159	
Disorganization symptoms	2.74 (2.14), 2.00	5.77 (3.37), 5.00		z=-3.505, *p* < 0.001, *r*=-0.163	
General Symptoms	5.90 (4.07), 6.00	10.77 (6.19), 12.00		z=-3.047, *p* = 0.002, *r*=-0.142	
Clusters					
Cluster 1, included, n (%)	111 (24.8%)		11 (84.6%)		χ²(1) = 23.168, *p* < 0.001; V = 0.224

**Table 4:** Cox regression models of conversion to psychoses

	Beta	Standard error	Wald (df = 1)		Significance	Exp(Beta)	95.0% Confidence interval (CI) of Exp(Beta)	
							lower	upper
Subscales, total sample^4^								
Positive symptoms	0.181	0.066	7.623		*p* = 0.006	1.199	1.054	1.364
Negative symptoms	0.152	0.058	6.802		*p* = 0.009	1.164	1.038	1.305
Clusters, total sample^5^								
Cluster 1 „high scorers“	3.098^1^	0.790	15.372		*p* < 0.001	22.159	4.709	104.276
Subscales, CHR-P sample^6^								
Disorganization symptoms	0.317	0.099	10.329		*p* = 0.001	1.373	1.132	1.666
Clusters, CHR-P sample^7^								
Cluster 1 „high scorers“	2.232^2^	1.052	4.504		*p* = 0.034	9.317	1.186	73.186
Subscales, UHR sample^8^								
Negative symptoms	0.220	0.064	11.978		*p* < 0.001	1.246	1.100	1.411
Neuroticism	-0.086	0.045	3.599		*p* = 0.058	0.918	0.840	1.003
SIPS subscales, UHR sample^9^								
Negative symptoms	0.163	0.058	7.920		*p* = 0.005	1.177	1.051	1.319
Clusters, UHR sample^10^								
Cluster 1 „high scorers“	3.712^3^	2.772	1.793		*p* = 0.181	40.954	0.179	9380.630

^1^ Bootstrap: *p* < 0.001; 95% CI (Beta): 2.018, 6.633

^2^ Bootstrap: *p* = 0.018; 95% CI (Beta): 0.037, 4.210

^3^ Bootstrap: *p* < 0.001; 95% CI (Beta): 3.439, 4.072

^4^ Model fit: χ²(2) = 37.252, *p* < 0.001

^5^ Model fit: χ²(1) = 29.248, *p* < 0.001

^6^ Model fit: χ²(1) = 10.790, *p* = 0.001

^7^ Model fit: χ²(1) = 6.553, *p* = 0.010

^8^ Model fit: χ²(2) = 13.598, *p* = 0.001

^9^ Model fit: χ²(1) = 8.766, *p* = 0.003

^10^ Model fit: χ²(1) = 5.441, *p* = 0.020

## Discussion

Negative symptoms (NS) significantly impair social functioning in schizophrenia and are linked to poorer outcomes like greater cognitive impairments, reduced quality of life and poor response to therapeutic interventions during adolescence [13, 30–33]. However, despite this recognized importance, NS are understudied in their specific contribution to the clinical presentation and outcomes in children and adolescents at clinical high-risk for psychosis (CHR-P). Thus, the present study examined the potential of subjective and attenuated NS according to the SPI-CY “Adynamia” and SIPS “Negative Symptoms” subscales to differentiate youth at CHR-P from clinical controls (CC) and predict psychotic conversion. Based on the SPI-CY pilot study by Fux et al. [24] and prediction study by Zhang et al. [25], we had hypothesized that these scales would best distinguish CHR-P from other clinical states and predict psychosis. However, the observed results only partially confirmed previous reports that subjective and attenuated NS best discriminate underage CHR-P patients and are the most important predictors of conversion to psychosis, respectively.

### Group discrimination by subjective negative symptoms

In contrast to the SPI-CY pilot study [24] (see sTable 7 for comparisons with our study), “Perception disturbances” and not “Adynamia” showed the largest group effect in our study. While both “Perception disturbances” and “Adynamia” had a median score that was four points higher than in the pilot study, the former had a larger and the latter a smaller effect size. Furthermore, the effect of “Adynamia” was also second to that of “Thought and motor disturbances”, whose effect size was similar to that found in the pilot study. The effect of “Neuroticism” was similarly moderate in both studies and discriminated the groups least.

The fourteen BS relevant to COPER and COGDIS are part of “Perception disturbances” and “Thought and motor disturbances”, whereby “Perception disturbances” is most relevant to COPER that includes four main items of this section compared to COGDIS that just includes one item. Because both studies have similar proportions of patients with COPER [24] (sTable 5), our higher median of “Perception disturbances” and relatedly the higher effect was not related to the proportion of COPER patients. Further, our larger proportion of COGDIS patients did not result in a larger median and effect of “Thought and motor disturbances” in our CHR-P. This indicates that the discriminatory effects of the two SPI-CY subscales that include criteria-relevant BS were not primarily related to the distribution of BS criteria.

Sociodemographic sample differences may explain the discrepancies in effect sizes for “Perception disturbances” and “Thought and motor disturbances” between our study and the pilot study [25]. Our sample was younger on average (by one year) and included more females (20% more in CHR-P and 10% more in CC) (sTable 7); both younger age and female sex were significantly associated with a higher prevalence of BS included in COGDIS and/or COPER in the community [11]. Thus, our higher proportion of females, particularly among CHR-P, as well as of younger patients might have increased the group effect of in “Perception disturbances” and “Thought and motor disturbances”. While this view was partly supported by the median of “Perception disturbances” in the CHR-P group that was twice as high as in the pilot study, yet equal in the CC group, our medians for “Thought and motor disturbances” were lower. Thus, no general influence of younger age and a higher proportion of girls in the sense of more or more severe perceptual and cognitive BS was observed in our sample.

However, age might have contributed indirectly to the median of “Thought and motor disturbances” in our CC group that included the highest rate of children, i.e., 8-12-year-olds. Five of the 19 items of this subscale cannot be assessed until the age of 13 years for developmental reasons [22] and score ‘0’ in children, thereby lowering its median. Thus, the higher number of children in our sample will have reduced the group effect of “Thought and motor disturbances” compared to the pilot group. Because of the relatively low number 8-12-year-olds, in particular in the CHR-P sample, that prevented targeted group comparison of the symptom profiles of only 8- to 12-year-old patients, larger, dedicated studies focusing on children are necessary to study very early, developmentally sensitive predictors of psychosis. So far, most paediatric CHR-P studies included 12-18- or 15-18-year-olds [34].

Despite larger group effects in “Perception disturbances” and “Thought and motor disturbances”, “Adynamia” emerged as the most important feature distinguishing the two clusters of high- and low-scorers, while “Negative symptoms” ranked fifth. The smaller Cluster 1 included a good quarter of high-scoring patients, mostly CHR-P, who were also significantly more impaired in functioning than Cluster 2 patients. While the higher functional impairment might be related to higher rates of mental disorders in Cluster 1, particularly of affective, anxiety, substance use, and stress-related disorders, the same pattern of differences in distribution of mental disorders was found at baseline between CHR-P and CC; yet without any indication of a group difference in functioning. Thus, this finding may better align with the idea that negative symptoms – in this case subtle, subclinical and self-experienced forms such as adynamic BS [14] – are a key domain of the psychosis spectrum in minors and strongly associated with functional deficits [13].

### Prediction of psychosis by subjective and attenuated negative symptoms

Supporting previous reports [16, 35], the subscale “Negative symptoms” was the sole predictor of conversion in our UHR sample when only SIPS subscales were considered as in Zhang et al. [25]. When all subscales were considered in the UHR sample, “Neuroticism” joined as a negative predictor, albeit one, only approaching significance. Some items of “Neuroticism” resemble borderline personality phenomena, such as irritability, increased emotional responsiveness and depersonalisation. Earlier studies had reported significant positive correlations of borderline symptoms with “Positive symptoms” but not with BS criteria or conversion to psychosis in UHR samples [36–38]. Thus, the slight negative psychosis-predictive value of “Neuroticism” might be mediated by the lower positive symptoms in the UHR patients.

In the total sample, “Negative symptoms” and “Positive symptoms” predicted conversion. This is in line with findings of Lindgren et al. [12] who compared the generalizability of 19 prediction models to clinical practice in an unselected adolescent psychiatric sample with a third fulfilling UHR criteria. Such a broader sample composition that also includes persons not meeting UHR criteria naturally results in a greater variance in “Positive symptoms” that defines the symptomatic UHR criteria. This resulted in a significant group effect with regard to conversion for “Positive symptoms” in our sample that was not found in the sample of Zhang et al. [25], although the means of “Positive symptoms” in our and Zhang’s converters are identical. The mean of “Negative symptoms” in our converters however was slightly lower than that of Zhang et al. [25] and clearly lower in the non-converters. Nevertheless, the significant predictive contributions of “Negative symptoms” in our and Zhang’s model were similarly low.

Interestingly, “Negative symptoms” had a lower group effect with regard to conversion than “Perception disturbances” and “Thought and motor disturbances” in group comparisons. Thus, the selection of “Negative symptoms” into the regression model indicates that there were too many redundancies between both “Perception disturbances” and “Thought and motor disturbances”, and “Positive symptoms”, in particular with regard to perception symptoms [39], that prevented the inclusion of these two BS-criteria-relevant subscales into the model in addition to “Positive symptoms”. These high redundancies between the criteria-relevant subscales of SPI-CY and SIPS, however, support the alternative use of UHR and BS criteria, albeit the two approaches reflecting distinct psychopathology. Such a redundancy seemed to be low between “Negative symptoms” and “Positive symptoms”, thus making NS a valuable addition in the early detection of psychosis in minors [12, 24, 25].

In the CHR-P sample, only the SIPS subscale “Disorganization symptoms” subscale, which notably not includes the positive item “disorganized communication”, was predictive of psychosis. This sole selection is surprising in light of its rather small group effect with regard to both initial group assignment and conversion. Yet, disorganized symptoms had been included in several prediction models, though always in concert with other symptoms and always in concert with positive symptoms [40]. The non-selection of “Positive symptoms” into the model on our CHR-P sample might be related to the fact that, of the 12 converters, all had met COPER and/or COGDIS at baseline but only 9 had also met symptomatic UHR criteria. Thus, the selection of “Disorganization symptoms” might reflect the presence of severe and observable “Trouble with focus and attention”, which is part of “Disorganization symptoms” and a result of severe cognitive BS but also the presence of more “Bizarre thinking” that dependent on the presence of unusual and delusion-like ideas. Though not related to conversion, “Disorganized symptoms” had also been related to cannabis use [41]; and indeed, substance use was more frequent in CHR-P converters (16.7%) compared to CHR-P non-converters (7.6%). This might have additionally contributed to our finding, but, just as single-item contributions, should be explored in more detail in future studies.

Regarding the cluster solution, unsurprisingly, the high-scorers’ Cluster 1 – which consisted mostly of CHR-P patients and all converters but the two with the longest time to conversion – was predictive of transition in Cox regression models, and, in pairwise comparisons, the cluster solution best distinguished converters from non-converters. Like the Cox regression solution, the cluster solution, which was primarily based on “Adynamia” and “Thought and motor disturbances”, indicated a combination of subscales representing both the positive and negative symptom domain to be most important in underage patients.

### Strengths and limitations

Key strengths of our study include the substantial sample size, the use of both UHR and BS criteria, the high control of data quality, and the inclusion of a CC group clinically unsuspected to develop psychosis but with primary diagnoses associated with increased risk for psychosis in adulthood and with symptom severity sufficient to require inpatient or at least day clinic treatment. While this high symptom severity might have biased the CC group towards higher symptom severity also on SPI-CY and SIPS, that this was not the case. Rather the CHR-P group comprising mostly outpatients showed the highest SIPS and SPI-CY subscale means overall, extending earlier reports from the same study that CHR-P criteria and symptoms are not merely a symptom severity marker or transdiagnostic phenomena [27]. Nevertheless, the small number of converters is a notable limitation, particularly given the recommended event-to-predictor ratio of 5:1 for reliable regression analyses [42].

Furthermore, our follow-up period of two years may have been too short, as psychoses with first prodromal symptoms before the age of 18 years show an extended prodrome [43]. At the same time, follow-up rates of 2 years are common in early detection studies, and most transitions happen in the first two years [8]. Another limitation is the dropout rate, which is significant in the CHR group, especially at the two-year follow-up. Nonetheless, dropouts and participants with follow-up data were largely similar, especially in clinical variables. While equating dropout with non-conversion likely reduced the discriminatory effects of subscales in pairwise comparisons, this limitation should have affected all subscales equally.

## Conclusion

Subclinical and attenuated NS in minors are largely neglected both in research and clinical practice. While our findings partly support an important role of subjective and attenuated NS in CHR-P and conversion to psychosis, they do not support an overall outstanding role. Rather, the role of NS seems to largely depend on sample composition and, with regard to conversion, was most pronounced in UHR samples. Future studies should therefore focus on differentiating NS profiles specifically within these populations. Furthermore, most specific interventions in underage UHR samples focused on positive symptoms, with only three studies so far having also analysed the effect on NS [44]. Our results indicate that the reduction of NS should be given more attention in future intervention trials, in particular in underage UHR samples.

## Supplementary Information

Below is the link to the electronic supplementary material.

Supplementary Material 1

Supplementary Material 2

## Data Availability

No datasets were generated or analysed during the current study.
