# Supplementary material for: Subclinical and attenuated negative symptoms in the prediction of psychosis in children and adolescents: sample composition matters
Source: Eur Child Adolesc Psychiatry. 2026 Jun 6;35(10):3145–55. doi: 10.1007/s00787-026-03042-2 (PMC13696592; doi:10.1007/s00787-026-03042-2)
Supplement: Supplementary file 1 [file 787_2026_3042_MOESM1_ESM.docx]

**sText 1. Description of attenuated and subjective negative symptoms as assessed with the Structured Interview for Psychosis-Risk Syndromes (SIPS) and the Schizophrenia Proneness Instrument, Child & Youth version (SPI-CY)**

The SIPS negative symptom items include:

- social anhedonia, ranging from ‘1’=patient is lightly socially awkward or anxious but socially active to ‘6’=patient has no friends and prefers being alone.
- avolition, ranging from ‘1’=patient has maintained focus on goal-directed activities but less than what would be considered average to ‘6’= patient does not participate in virtually any goal-directed activities with prodding of others to do so being unsuccessful.
- expression of emotion, ranging from ‘1’=patient’s emotional responsiveness is slightly delayed or blunted to ‘6’= patient has flat affect, monotone speech, and is unable to become involved with interviewer or maintain conversation despite active questioning by the interviewer
- experience of emotions and self, ranging from ‘1’=patient reports feeling distant from others and experiencing everyday feelings as muted to ‘6’=patient reports feeling profoundly changed and possibly alien to self, a feeling of no feelings
- ideational richness, ranging from ‘1’=some conversational awkwardness observable to ‘6’=patient is unable, at times, to follow any conversation no matter how simple and shows a verbal content and expression that is mostly limited to single words and yes/no responses.
- occupational functioning, ranging from ‘1’=more than average effort and focus required from the patient to maintain usual level of performance at work, school to ‘6’=patient failed or left school, left employment or was fired.

Basic symptoms have to be subjectively experienced by the young person as new and/or different from ‘normal’ troubles. These symptoms should have been absent, or only present at a significantly lower frequency, in what the young person considers his or her premorbid stage. Thus, a symptom is scored only when experienced by the young person as a disturbance, deficiency or complaint, irrespective of its appearance in behavior, gesture, miming or speech and when the possibility that it is an effect of drug use, especially of cannabis or ketamine, a side-effect of medication, a manifestation of a somatic illness or a by-product of a hypnagogic / hypnopompic state was excluded. The SPI-CY “Anhedonia” section includes the following symptoms:

- Reduced energy and vitality
- Reduced persistence and patience
- Reduced drive and initiative
- Impaired tolerance to physical and/or mental labor
- Impaired tolerance to unusual, unexpected or specific novel demands
- Impaired tolerance to certain social everyday situations
- Impaired tolerance to working under pressure of time or rapidly changing different demands
- Change in mood and emotional responsiveness
- Decrease in positive emotional responsiveness towards others
- Intermittent, recurrent depressive mood swings
- Disturbance in presenting oneself in terms of a disturbance in the ability to express oneself non-verbally.
- Increased emotional reactivity in response to everyday events
- Increased emotional reactivity in response to routine social interactions that affect the young person directly or indirectly
- Difficulties concentrating in terms of maintaining attention
- Forgetfulness, scatterbrainedness in terms of deficits in implicit (non-declarative), i.e., unconscious, unintentional memory
- Slowed-down thinking
- Lack of ‘thought energy’ or goal-directed thoughts

## sText 2. Additional information on the recruitment of samples.

**Clinical high-risk for psychosis (CHR-P) sample**: The CHR-P sample was recruited at all three sites (Bern, Switzerland; Zurich, Switzerland; and Cologne, Germany), mainly from the clientele of established local early detection services. These services accepted both outpatients and inpatients who were clinically suspected of having psychosis and who were referred by (mental) health professionals, as well as individuals who approached the service themselves out of fear of developing psychosis.

**Clinical control (CC) sample**: The CC sample was also recruited at all three sites from the inpatients with the primary diagnoses relevant for inclusion but no clinical suspicion of emerging psychoses.

**Community sample**: The community sample was exclusively recruited in Bern, Switzerland. For the recruitment of a representative community sample, the Agency for Informatics and Organization of the Canton Bern randomly drew a sample (including addresses) stratified for age and sex from the population register of the city of Bern and its urban hinterland (approximately 200,000 residents). Subsequently, we searched directories and the Internet for telephone numbers. The availability of a working telephone number served as an eligibility criterion in this group. We established first contact by an information letter, personally addressing each potential participant and his/her parents. Next, we contacted parents and/or their children by phone, informed them in detail and asked them to give written informed consent and assent, respectively. In children below age 16.0 years, we contacted parents first.

N = 980 persons were drawn from the register, for n = 176 of them, we could not ascertain a working telephone number and n = 41 persons were drawn twice. Of the remaining n = 763 persons, n = 234 agreed to participate, yet one person later on withdrew consent. n = 353 did not agree, mainly for lack of interest (35.6%) or time (35.9%). Further, we excluded n = 52 persons because they had turned 18 years by the time contact was made (53.9%), had moved away from the greater Bern area (32.7%), or lacked the ability to participate in the study for language or physical health reasons (13.5%). With n = 124 persons, all attempts (at least 40) to reach them on the phone remained fruitless.

Thus, according to the standard definitions of the American Association for Public Opinion Research (2016), the contact rate was 82.7%, the cooperation rate was 39.9%, the refusal rate was 49.2%, and the response rate was 32.6%.

**References**:

American Association for Public Opinion Research. Standard Definitions Final Dispositions of Case Codes and Outcome Rates for Surveys. 2016. [cited 20 February 2021]. Available from: http://www.aapor.org/AAPOR_Main/media/publications/Standard-Definitions20169theditionfinal.pdf.

## sText 3. Reasons for rating patients lost-to-follow-up as non-converters.

The decision to rate patients with missing follow-up conservatively as non-converters was made for four reasons:

- At statistical level, we wanted to maintain sufficient power;
- Meta-analyses of conversion suggest a conversion rate in CHR-P around 20% within 2 years (for example: Schultze-Lutter et al., 2015; Fusar-Poli P et al., 2016; Salazar de Pablo et al., 2021), thus, for any CHR-P patient and even more for any CC inpatient (CHR-P negative patients have a conversion rate around 1%; see references above) who could not be followed up, a non-conversion was far more likely than a conversion.
- Because all three early detection services were part of a larger child and adolescent psychiatric university hospital serving the larger area of the Canton Bern, the city of Zurich and the city of Cologne, even lost-to-follow-up patients who had developed a first episode of psychosis would very likely have come to our attention.
- In all cases in which a follow-up could not be conducted with the patient but contact was made with a parent, it was reported that the mental health status of the patient had much increased and refusal was due to not wanting to look back and wanting to forget about the phase of mental ill health

**References:**

Fusar-Poli P, et al. Heterogeneity of Psychosis Risk Within Individuals at Clinical High Risk: A Meta-analytical Stratification. JAMA Psychiatry. 2016;73(2):113-20. doi: 10.1001/jamapsychiatry.2015.2324

Salazar de Pablo G, et al. Probability of Transition to Psychosis in Individuals at Clinical High Risk: An Updated Meta-analysis. JAMA Psychiatry. 2021;78(9):970-978. doi: 10.1001/jamapsychiatry.2021.0830

Schultze-Lutter F, et al. EPA guidance on the early detection of clinical high risk states of psychoses. Eur Psychiatry. 2015r;30(3):405-16. doi: 10.1016/j.eurpsy.2015.01.010.

## sFigure 1. Recruitment and follow-up of the analysed sample (N=460).

CHR-P: clinical high-risk for psychosis; EAT: eating disorders (Anorexia or Bulimia nervosa); ADHD: attention deficit/hyperactivity disorders; AD/OCD: anxiety (social or specific phobia) and obsessive-compulsive disorders; AS: Asperger’s disorder

The primary diagnoses of the clinical control sample were chosen according to the prospective study of Rubino et al. (2009) that associated the following diagnoses in childhood and adolescence with a higher frequency of psychosis in adulthood: attention deficit/hyperactivity disorders, anxiety (social or specific phobia) and obstetric-compulsive disorders and eating disorders (Anorexia or Bulimia nervosa). This study did not include patients with Asperger’s disorder; but as this condition was recognized as a condition with increased risk to develop psychosis in young adulthood (APA, 1994), we included this condition as a primary diagnosis in childhood and adolescence with a higher likelihood of psychosis in adulthood in addition.

**References**:

American Psychiatric Association. (1994). Diagnostic and statistical manual of mental disorders (4th ed.). American Psychiatric Publishing, Inc..

Rubino IA, Frank E, Croce Nanni R, Pozzi D, Lanza di Scalea T, Siracusano A. A comparative study of axis I antecedents before age 18 of unipolar depression, bipolar disorder and schizophrenia. Psychopathology. 2009;42(5):325-32. doi: 10.1159/000232975

**sTable 1: Criteria of a clinical high-risk for psychosis (CHR-P) according to the Schizophrenia Proneness Instrument, Child & Youth version (SPI-CY) and the Structured Interview for Psychosis-Risk Syndromes (SIPS)**

**1a. The basic symptom criteria “Cognitive Disturbances” (COGDIS) and “Cognitive-Perceptive BS” (COPER)**

**“Cognitive Disturbances” (COGDIS)**

≥2 basic symptoms of at least weekly frequency (SPI ≥ ‘3‘) within past 3 months:

- inability to divide attention
- thought interference
- thought pressure
- thought blockages
- disturbance of receptive speech
- disturbance of expressive speech
- disturbance of abstract thinking
- unstable ideas of reference
- captivation of attention by details of the visual field

**“Cognitive-Perceptive BS” (COPER)**

≥1 basic symptom of at least weekly frequency (SPI ≥ ’3‘) within past 3 months and first occurrence or significant increase in frequency at least 12 months ago:

- thought interference
- thought perseveration
- thought pressure
- thought blockages
- dist. of receptive speech
- decreased ability to discriminate between ideas and perception, fantasy and real memories
- unstable ideas of reference
- derealisation
- visual perception disturbances (14 subitems)
- acoustic perception disturbances (3 subitems)

**1b. The ultra-high risk (UHR) criteria “Attenuated Psychosis Symptoms Syndrome” (APSS), “Brief Intermittent Psychotic Symptoms Syndrome” (BIPSS) and “Genetic Risk and Functional Decline” (GRFD)**

**“Attenuated Psychosis Symptoms Syndrome” (APSS)**

≥1 positive symptom with a score of ‘3’-‘5’ that (I) is not better explained by another mental disorder, (II) has begun within the past year or do any currently rate one or more scale points higher compared to 12 months ago and (III) occurred at an average frequency of at least once per week in the past month:

- unusual thought content/delusional ideas
- suspiciousness/persecutory ideas
- grandiosity
- perceptual abnormalities/hallucinations
- disorganized communication

**“Brief Intermittent Psychotic Symptoms Syndrome” (BIPSS)**

≥1 positive symptom (see above) with a score of ‘6’ that (I) is not better explained by another mental disorder, (II) has reached a psychotic level of intensity in the past three months, and (III) is currently present for at least several minutes per day at a frequency of at least once per month but less than 7 days.

**Genetic Risk and Functional Decline” (GRFD)**

The patient (I) meets criteria for Schizotypal Personality Disorder or has a first degree relative with a psychotic disorder and (II) is experiencing at least a 30% drop in GAF score over the last month as compared to 12 months ago.

## sTable 2. Baseline characteristics of CHR-P patients with and without any follow-up (N=183).

|  | **With follow-up (n=147, 80.3%)** | **Without follow-up (n=36, 19.7%)** | **Statistics, effect size** |
| --- | --- | --- | --- |
| **Age**, mean (SD), median | 15.06 (2.11), 15.45 | 16.29 (1.56), 16.35 | z=-3.460, p<0.001; r=-0.256 |
| **Sex**, male, n (%) | 50 (34.0%) | 19 (52.8%) | χ²(1)=4.335, p=0.037; V=0.154 |
| **Nationality**, n (%) |  |  | χ²(2)=11.191, p=0.004; V=0.247 |
| Switzerland | 73 (49.7%) | 29 (80.6%) |  |
| Germany | 52 (35.4%) | 5 (13.9%) |  |
| Other | 22 (15.0%) | 2 (5.6%) |  |
| **Highest achieved/targeted school graduation**, n (%) |  |  | χ²(3)=11.279, p=0.010; V=0.248 |
| ISCED 0 | 0 | 2 (5.6%) |  |
| ISCED 1 | 20 (13.6%) | 1 (2.8%) |  |
| ISCED 2 | 70 (47.6%) | 19 (52.8%) |  |
| ISCED 3 | 57 (38.8%) | 14 (38.8%) |  |
| **COGDIS**, present, n (%)^a^ | 64 (43.5%) | 17 (47.2%) | χ²(1)=0.159, p=0.690; V=0.029 |
| **COPER**, present, n (%)^a^ | 88 (59.9%) | 26 (72.2%) | χ²(1)=1.880, p=0.170; V=0.101 |
| **APSS**, present, n (%)^a^ | 91 (61.9%) | 21 (58.3%) | χ²(1)=0.155, p=0.693; V=0.029 |
| **BIPSS**, present, n (%)^a^ | 4 (2.7%) | 1 (2.8%) | χ²(1)<0.001, p=1.000; V=0.001 |
| **GRFD**, present, n (%) | 8 (5.4%) | 2 (5.6%) | χ²(1)=0.001, p=1.000; V=0.002 |
| **Any affective disorders**, present, n (%)^a,b^ | 58 (39.5%) | 11 (30.6%) | χ²(1)=0.975, p=0.323; V=0.073 |
| **Any anxiety disorders**, present, n (%)^a,b^ | 65 (44.2%) | 10 (27.8%) | χ²(1)=3.231, p=0.072; V=0.133 |
| **Any obsessive-compulsive disorders**, present, n (%)^a,b^ | 13 (8.8%) | 2 (5.6%) | χ²(1)=0.415, p=0.519; V=0.048 |
| **Any eating disorders, present**, n (%)^a,b^ | 13 (8.8%) | 3 (8.3%) | χ²(1)=0.009, p=1.000; V=0.007 |
| **Any somatoform disorders**, present, n (%)^a,b^ | 3 (2.0%) | 0 | χ²(1)=0.747, p=1.000; V=0.064 |
| **Any substance misuse**, present, n (%)^a,b^ | 10 (6.8%) | 5 (13.9%) | χ²(1)=1.930, p=0.165; V=0.103 |
| **Any trauma/stress-related disorders**, present, n (%)^a,b^ | 11 (7.5%) | 2 (5.6%) | χ²(1)=0.163, p=1.000; V=0.030 |
| **Any developmental disorders**, present, n (%)^a,b^ | 20 (13.6%) | 5 (13.9%) | χ²(1)=0.002, p=1.000; V=0.003 |
| **SOFAS**, mean (SD), median | 60.14 (12.62), 60.0 | 57.75 (9.50), 59.5 | z=-0.832, p=0.410; r=-0.062 |
| **Adynamia**, mean (SD), median | 29.90 (20.81), 29.0 | 27.33 (17.07), 25.5 | z=-0.615, p=0.539; r=-0.045 |
| **Perception disturbance**, mean (SD), median **s** | 11.80 (13.10), 8.0 | 7.33 (7.69), 6.0 | z=-1.467, p=0.142; r=-0.108 |
| **Neuroticism**, mean (SD), median | 9.82 (7.41), 9.0 | 8.58 (7.15), 7.0 | z=-0.936, p=0.349; r=-0.069 |
| **Thought and motor disturbances**, mean (SD), median | 14.37 (12.90), 13.0 | 13.19 (11.57), 12.0 | z=-0.364, p=0.716; r=-0.027 |
| **Positive symptoms**, mean (SD), median | 8.53 (4.18), 9.0 | 7.75 (4.67), 7.0 | z=-0.942, p=0.346; r=-0.070 |
| **Negative symptoms**, mean (SD), median | 9.49 (5.80), 10.0 | 11.42 (5.82), 10.0 | z=-1.672, p=0.094; r=-0.124 |
| **Disorganization symptoms**, mean (SD), median | 3.92 (2.60), 4.0 | 3.69 (2.21), 3.0 | z=-0.461, p=0.645; r=-0.034 |
| **General symptoms**, mean (SD), median | 8.46 (4.48), 9.0 | 7.53 (3.42), 8.0 | z=-1.710, p=0.087; r=-0.126 |
| **Cluster 1**, present, n (%) | 89 (60.5%) | 17 (47.2%) | χ²(1)=2.106, p=0.147; V=0.107 |

^a^ multiple CHR-P criteria and diagnosis possible

^b^ assessed with the Mini-International Neuropsychiatric Interview for Children and Adolescents (M.I.N.I. Kid) (Sheehan, et al., 2010).

ISCED: International Standard Classification of Education 2011; CHR-P: clinical high-risk for psychosis; COGDIS: Cognitive Disturbances; COPER: Cognitive-Perceptive basic symptoms; APSS: attenuated psychotic symptoms syndrome; BIPSS: brief intermittent psychotic symptoms syndrome; GRFD: genetic risk and functional decline; SOFAS: Social and Occupational Functioning Assessment Scale (0=lowest to 100=highest functioning)

**Reference**: Sheehan DV, Sheehan KH, Shytle RD, et al. Reliability and validity of the Mini International Neuropsychiatric Interview for Children and Adolescents (MINI-KID). J Clin Psychiatry. 2010;71(3):313-26. doi: 10.4088/JCP.09m05305whi.

## sTable 3. Baseline characteristics of clinical controls with and without any follow-up (N=277).

|  | **With follow-up (n=230, 83.0%)** | **Without follow-up (n=47, 17.0%)** | **Statistics, effect size** |
| --- | --- | --- | --- |
| **Age**, mean (SD), median | 14.23 (2.59), 14.81 | 14.42 (2.30), 14.88 | z=-0.236, p=0.814; r=-0.014 |
| **Sex**, male, n (%) | 107 (46.5%) | 19 (40.4%) | χ²(1)=0.585, p=0.444; V=0.046 |
| **Nationality**, n (%) |  |  | χ²(2)=1.710, p=0.425.; V=0.079 |
| Switzerland | 123 (53.5%) | 30 (63.8%) |  |
| Germany | 93 (40.4%) | 15 (31.9%) |  |
| Other | 14 (6.1%) | 2 (4.3%) |  |
| **Highest achieved/targeted school graduation**, n (%) |  |  | χ²(3)=9.239, p=0.026; V=0.183 |
| ISCED 0 | 1 (0.4%) | 0 |  |
| ISCED 1 | 61 (26.5%) | 8 (17.0%) |  |
| ISCED 2 | 87 (37.8%) | 29 (61.7%) |  |
| ISCED 3 | 81 (35.2%) | 10 (21.3%) |  |
| **Any affective disorders**, present, n (%)^a,b^ | 37 (16.1%) | 6 (12.8%) | χ²(1)=0.328, p=0.567; V=0.034 |
| **Any anxiety disorders**, present, n (%)^a,b^ | 60 (26.1%) | 12 (25.5%) | χ²(1)=0.006, p=0.937; V=0.005 |
| **Any obsessive-compulsive disorders**, present, n (%)^a,b^ | 29 (12.6%) | 2 (4.3%) | χ²(1)=2.740, p=0.098; V=0.099 |
| **Any eating disorders, present**, n (%)^a,b^ | 66 (28.7%) | 20 (42.6%) | χ²(1)=3.501, p=0.061; V=0.112 |
| **Any somatoform disorders**, present, n (%)^a,b^ | 3 (1.3%) | 0 | χ²(1)=0.620, p=0.431; V=0.047 |
| **Any substance misuse**, present, n (%)^a,b^ | 3 (1.3%) | 1 (2.1%) | χ²(1)=0.186, p=0.527; V=0.026 |
| **Any trauma/stress-related disorders**, present, n (%)^a,b^ | 2 (0.9%) | 0 | χ²(1)=0.412, p=1.000; V=0.039 |
| **Any developmental disorders**, present, n (%)^a,b^ | 103 (44.8%) | 19 (40.4%) | χ²(1)=0.301, p=0.583; V=0.033 |
| **SOFAS**, mean (SD), median | 60.33 (10.80), 60.0 | 59.98 (12.60), 58.0 | z=-0.522, p=0.601; r=-0.031 |
| **Adynamia**, mean (SD), median | 11.92 (13.61), 8.0 | 10.98 (11.01), 8.0 | z=-0.024, p=0.981; r=-0.001 |
| **Perception disturbance**, mean (SD), median **s** | 1.20 (2.68), 0.0 | 0.96 (2.18), 0.0 | z=-0.729, p=0.466; r=-0.044 |
| **Neuroticism**, mean (SD), median | 4.48 (4.85), 3.0 | 4.74 (4.42), 4.0 | z=-0.683, p=0.495; r=-0.041 |
| **Thought and motor disturbances**, mean (SD), median | 3.54 (5.24), 0.5 | 2.83 (3.47), 1.0 | z=-0.012, p=0.991; r=-0.001 |
| **Positive symptoms**, mean (SD), median | 2.30 (2.33), 1.0 | 1.68 (1.86), 1.0 | z=-1.581, p=0.114; r=-0.095 |
| **Negative symptoms**, mean (SD), median | 5.13 (4.08), 4.5 | 6.13 (4.71), 5.0 | z=-1.223, p=0.221; r=-0.073 |
| **Disorganization symptoms**, mean (SD), median | 2.13 (1.76), 2.0 | 2.13 (1.38), 2.0 | z=-0.430, p=0.667; r=-0.026 |
| **General symptoms**, mean (SD), median | 4.43 (3.32), 4.0 | 5.19 (3.95), 5.0 | z=-1.018, p=0.309; r=-0.061 |
| **Cluster 1**, present, n (%) | 14 (6.1%) | 2 (4.3%) | χ²(1)=0.241, p=0.624; V=0.029 |

^a^ multiple CHR-P criteria and diagnosis possible

^b^ assessed with the Mini-International Neuropsychiatric Interview for Children and Adolescents (M.I.N.I. Kid) (Sheehan, et al., 2010).

ISCED: International Standard Classification of Education 2011; SOFAS: Social and Occupational Functioning Assessment Scale (0=lowest to 100=highest functioning)

## sTable 4: Discriminating patient groups: Receiver Operating Characteristic (ROC) analyses of subscales

|  | **Area under the curve** | **Standard error** | **Asymptotic significance** | **95% Confidence interval** | |
| --- | --- | --- | --- | --- | --- |
|  |  |  |  | **lower** | **upper** |
| **Adynamia** | 0.765 | 0.024 | p<0.001 | 0.719 | 0.811 |
| **Perception disturbances** | 0.835 | 0.021 | p<0.001 | 0.795 | 0.876 |
| **Neuroticism** | 0.708 | 0.025 | p<0.001 | 0.659 | 0.758 |
| **Thought and motor disturbances** | 0.786 | 0.023 | p<0.001 | 0.741 | 0.831 |
| **Positive symptoms** | 0.904 | 0.014 | p<0.001 | 0.876 | 0.932 |
| **Negative symptoms** | 0.731 | 0.025 | p<0.001 | 0.683 | 0.780 |
| **Disorganization symptoms** | 0.703 | 0.025 | p<0.001 | 0.653 | 0.753 |
| **General symptoms** | 0.751 | 0.024 | p<0.001 | 0.703 | 0.799 |

Interpretation according to the review of de Hond et al. (2022): 0.7 ≤ area under the curve (AUC) < 0.8 = moderate; 0.8 ≤ AUC < 0.9 = good; 0.9 ≤ AUC = excellent

**Reference:** de Hond AAH, Steyerberg EW, van Calster B. Interpreting area under the receiver operating characteristic curve. Lancet Digit Health. 2022 Dec;4(12):e853-e855. doi: 10.1016/S2589-7500(22)00188-1.

## sTable 5. Baseline characteristics of clusters (N=460).

|  | **Cluster 1 (n=122, 26.5%)** | **Cluster 2 (n=338, 73.5%)** | **Statistics, effect size** |
| --- | --- | --- | --- |
| **Age**, mean (SD), median | 15.83 (1.49), 15.84 | 14.26 (2.55), 14.87 | z=-5.902, p<0.001; r=-0.275 |
| **Sex**, male, n (%) | 36 (29.5%) | 159 (47.0%) | χ²(1)=11.284, p<0.001; V=0.157 |
| **Nationality**, n (%) |  |  | χ²(2)=14.066, p<0.001.; V=0.175 |
| Switzerland | 51 (41.8%) | 204 (60.4%) |  |
| Germany | 54 (44.3%) | 111 (32.8%) |  |
| Other | 17 (13.9%) | 23 (6.8%) |  |
| **Highest achieved/targeted school graduation**, n (%) |  |  | χ²(3)=36.056, p<0.001; V=0.280 |
| ISCED 0 | 0 (0.0%) | 3 (0.9%) |  |
| ISCED 1 | 2 (1.6%) | 88 (26.0%) |  |
| ISCED 2 | 64 (52.5%) | 141 (41.7%) |  |
| ISCED 3 | 56 (45.9%) | 106 (31.4%) |  |
| **Clinical setting, n (%)** |  |  | χ²(1)=79.262, p<0.001; V=0.415 |
| Inpatient/day clinic | 55 (45.1%) | 290 (85.8%) |  |
| Outpatient | 67 (54.9%) | 48 (14.2%) |  |
| **Any CHR-P criteria**, present, n (%) | 106 (86.9%) | 77 (22.8%) | χ²(1)=153.772, p<0.001; V=0.578 |
| **COGDIS**, present, n (%)^a^ | 65 (53.3%) | 16 (4.7%) | χ²(1)=145.612, p<0.001; V=0.563 |
| **COPER**, present, n (%)^a^ | 72 (59.0%) | 42 (12.4%) | χ²(1)=104.387, p<0.001; V=0.476 |
| **APSS**, present, n (%)^a^ | 78 (63.9%) | 34 (10.1%) | χ²(1)=141.259, p<0.001; V=0.554 |
| **BIPSS**, present, n (%)^a^ | 3 (2.5%) | 2 (0.6%) | χ²(1)=2.907, p=0.119; V=0.079 |
| **GRFD**, present, n (%) | 3 (2.5%) | 7 (2.1%) | χ²(1)=0.063, p=0.729; V=0.012 |
| **Any affective disorders**, present, n (%)^a,b^ | 71 (58.2%) | 41 (12.1%) | χ²(1)=103.278, p<0.001; V=0.474 |
| **Any anxiety disorders**, present, n (%)^a,b^ | 59 (48.4%) | 88 (26.0%) | χ²(1)=20.548, p<0.001; V=0.211 |
| **Any obsessive-compulsive disorders**, present, n (%)^a,b^ | 12 (9.8%) | 34 (10.1%) | χ²(1)=0.005, p=0.994; V=0.003 |
| **Any eating disorders, present**, n (%)^a,b^ | 12 (9.8%) | 90 (26.6%) | χ²(1)=14.646, p<0.001; V=0.178 |
| **Any somatoform disorders**, present, n (%)^a,b^ | 2 (1.6%) | 4 (1.2%) | χ²(1)=0.145, p=0.658; V=0.018 |
| **Any substance misuse**, present, n (%)^a,b^ | 12 (9.8%) | 7 (2.1%) | χ²(1)=13.650, p<0.001; V=0.172 |
| **Any trauma/stress-related disorders**, present, n (%)^a,b^ | 9 (7.4%) | 6 (1.8%) | χ²(1)=8.918, p=0.006; V=0.139 |
| **Any developmental disorders**, present, n (%)^a,b^ | 18 (14.8%) | 129 (38.2%) | χ²(1)=22.596, p<0.001; V=0.222 |
| **SOFAS**, mean (SD), median | 55.06 (0.09), 54.00 | 61.82 (11.75), 60.00 | z=-5.648, p<0.001; r=-0.263 |

^a^ multiple CHR-P criteria and diagnosis possible

^b^ assessed with the Mini-International Neuropsychiatric Interview for Children and Adolescents (M.I.N.I. Kid) (Sheehan, et al., 2010).

ISCED: International Standard Classification of Education 2011; CHR-P: clinical high-risk for psychosis; COGDIS: Cognitive Disturbances; COPER: Cognitive-Perceptive basic symptoms; APSS: attenuated psychotic symptoms syndrome; BIPSS: brief intermittent psychotic symptoms syndrome; GRFD: genetic risk and functional decline; SOFAS: Social and Occupational Functioning Assessment Scale (0=lowest to 100=highest functioning)

## sTable 6: Overview of baseline characteristics and type of psychosis in converters (N=13)

|  | **Age** in yrs. | **Sex** | **Axis-I disorder** | **CHR-P criteria** | **Type of psychosis** | **Conversion time** in months | **Cluster** |
| --- | --- | --- | --- | --- | --- | --- | --- |
| **1** | 15 | Female | Developmental disorder | COPER, APSS | Delusional disorder | 2.30 | 1 |
| **2** | 15 | Female | None | COGDIS, APSS | Brief psychotic episode | 3.45 | 1 |
| **3** | 15 | Male | None | COGDIS, GRFD | Schizophrenia | 4.14 | 1 |
| **4** | 17 | Male | Substance misuse | COGDIS, APSS | Delusional disorder | 4.63 | 1 |
| **5** | 16 | Male | Affective disorder, anxiety disorder, developmental disorder | COGDIS, APSS | Schizophrenia | 5.42 | 1 |
| **6** | 14 | Female | Affective disorder, anxiety disorder, trauma/stress-related disorder | COGDIS, COPER, APSS | Major depression with psychotic symptoms | 7.36 | 1 |
| **7** | 16 | Male | Affective disorder, obsessive-compulsive disorder | COGDIS, COPER, APSS | Brief psychotic episode | 10.58 | 1 |
| **8** | 16 | Male | Anxiety disorder | COGDIS, COPER, APSS | Schizophrenia | 12.00 | 1 |
| **9** | 15 | Female | Affective disorder, substance misuse | COPER, APSS | Schizophrenia | 12.00 | 1 |
| **10** | 17 | Male | Affective disorder, developmental disorder | COGDIS | Major depression with psychotic symptoms | 12.78 | 1 |
| **11** | 11 | Male | Affective disorder, developmental disorder | COGDIS, APSS | Major depression with psychotic symptoms | 26.51 | 1 |
| **12** | 17 | Female | Anxiety disorder | COPER | Major depression with psychotic symptoms | 29.73 | 2 |
| **13** | 13 | Female | Developmental disorder | None | Schizoaffective disorder | 38.11 | 2 |

COGDIS: Cognitive Disturbances; COPER: Cognitive-Perceptive basic symptoms; APSS: attenuated psychotic symptoms syndrome; GRFD: genetic risk and functional decline

## sTable 7: Comparison between data of the SPI-CY pilot study by Fux et al. (2013) and the present study

|  | **Pilot study** | | **Present study** | |
| --- | --- | --- | --- | --- |
|  | **Effect sizes (Rosenthal’s r)** | | | |
| **Adynamia** | 0.70 | | 0.450 | |
| **Perception disturbances** | 0.41 | | 0.606 | |
| **Neuroticism** | 0.37 | | 0.356 | |
| **Thought and motor disturbances** | 0.53 | | 0.496 | |
|  | **Median subscale scores** | | | |
|  | ***CHR-P*** | ***Clinical controls*** | ***CHR-P*** | ***Clinical controls*** |
| **Adynamia** | 23 | 8 | 27 | 8 |
| **Perception disturbances** | 4 | 0 | 8 | 0 |
| **Neuroticism** | 7 | 2 | 9 | 3 |
| **Thought and motor disturbances** | 13 | 3 | 12 | 1 |
|  | **Discussed sociodemographic and clinical characteristics** | | | |
|  | ***CHR-P*** | ***Clinical controls*** | ***CHR-P*** | ***Clinical controls*** |
| **COPER**, present (%) | 65% | - | 62.3% | - |
| **COGDIS**, present (%) | 26% | - | 44.3% | - |
| **Sex**, male, present (%) | 57% | 55% | 37.7% | 45.5% |
| **Age**, mean (SD) | 16.8 (1.6) | 15.5 (2.3) | 15.30 (2.07) | 14.26 (2.54) |

CHR-P: clinical high-risk for psychosis; COGDIS: Cognitive Disturbances; COPER: Cognitive-Perceptive basic symptoms

**Reference**: Fux L, Walger P, Schimmelmann BG, Schultze-Lutter F (2013). The Schizophrenia Proneness Instrument, Child and Youth version (SPI-CY): practicability and discriminative validity. Schizophr Res. 146(1-3):69-78. doi: 10.1016/j.schres.2013.02.014.

## sTable 8: STROBE Statement for cohort studies

|  | Item No | Recommendation | Page No | |  |
| --- | --- | --- | --- | --- | --- |
| **Title and abstract** | 1 | (*a*) Indicate the study’s design with a commonly used term in the title or the abstract | 3 | |  |
|  |  | (*b*) Provide in the abstract an informative and balanced summary of what was done and what was found |  |  |  |
| Introduction | | | | | |
| Background/rationale | 2 | Explain the scientific background and rationale for the investigation being reported | 4 | |  |
| Objectives | 3 | State specific objectives, including any prespecified hypotheses | 4-5 | |  |
| Methods | | | | | |
| Study design | 4 | Present key elements of study design early in the paper | 5 | |  |
| Setting | 5 | Describe the setting, locations, and relevant dates, including periods of recruitment, exposure, follow-up, and data collection | 5 | |  |
| Participants | 6 | (*a*) Give the eligibility criteria, and the sources and methods of selection of participants. Describe methods of follow-up | 5 | |  |
|  |  | (*b*) For matched studies, give matching criteria and number of exposed and unexposed | Not applicable | |  |
| Variables | 7 | Clearly define all outcomes, exposures, predictors, potential confounders, and effect modifiers. Give diagnostic criteria, if applicable | 5 | |  |
| Data sources/ measurement | 8* | For each variable of interest, give sources of data and details of methods of assessment (measurement). Describe comparability of assessment methods if there is more than one group | 5-6 | |  |
| Bias | 9 | Describe any efforts to address potential sources of bias | 5 | |  |
| Study size | 10 | Explain how the study size was arrived at | 5 | |  |
| Quantitative variables | 11 | Explain how quantitative variables were handled in the analyses. If applicable, describe which groupings were chosen and why | 5-6 | |  |
| Statistical methods | 12 | (*a*) Describe all statistical methods, including those used to control for confounding | 5-6 | |  |
|  |  | (*b*) Describe any methods used to examine subgroups and interactions |  |  |  |
|  |  | (*c*) Explain how missing data were addressed |  |  |  |
|  |  | (*d*) If applicable, explain how loss to follow-up was addressed |  |  |  |
|  |  | (*e*) Describe any sensitivity analyses |  |  |  |
| Results | | |  | |  |
| Participants | 13* | (a) Report numbers of individuals at each stage of study—eg numbers potentially eligible, examined for eligibility, confirmed eligible, included in the study, completing follow-up, and analysed | sFigure 1 | |  |
|  |  | (b) Give reasons for non-participation at each stage |  |  |  |
|  |  | (c) Consider use of a flow diagram |  |  |  |
| Descriptive data | 14* | (a) Give characteristics of study participants (eg demographic, clinical, social) and information on exposures and potential confounders | 6,  Table 1, sTables 1-2 | |  |
|  |  | (b) Indicate number of participants with missing data for each variable of interest |  |  |  |
|  |  | (c) Summarise follow-up time (eg, average and total amount) |  |  |  |
| Outcome data | 15* | Report numbers of outcome events or summary measures over time | 6-7 | |  |
| Main results | 16 | (*a*) Give unadjusted estimates and, if applicable, confounder-adjusted estimates and their precision (eg, 95% confidence interval). Make clear which confounders were adjusted for and why they were included | 6-7,  Figure 1, Tables 2-4 |  |  |
|  |  | (*b*) Report category boundaries when continuous variables were categorized |  |  |  |
|  |  | (*c*) If relevant, consider translating estimates of relative risk into absolute risk for a meaningful time period |  |  |  |
| Other analyses | 17 | Report other analyses done—eg analyses of subgroups and interactions, and sensitivity analyses | 6-7 |  |  |
| Discussion | | | | | |
| Key results | 18 | Summarise key results with reference to study objectives | 7 |  |  |
| Limitations | 19 | Discuss limitations of the study, taking into account sources of potential bias or imprecision. Discuss both direction and magnitude of any potential bias | 10 |  |  |
| Interpretation | 20 | Give a cautious overall interpretation of results considering objectives, limitations, multiplicity of analyses, results from similar studies, and other relevant evidence | 7-10 |  |  |
| Generalisability | 21 | Discuss the generalisability (external validity) of the study results | 10 |  |  |
| Other information | | | | | |
| Funding | 22 | Give the source of funding and the role of the funders for the present study and, if applicable, for the original study on which the present article is based | 2 |  |  |
